# Design of the conserved epitope peptide of SARS-CoV-2 spike protein as the broad-spectrum COVID-19 vaccine

**DOI:** 10.1007/s00253-024-13331-y

**Published:** 2024-10-16

**Authors:** Ting-Yu Chang, Chia-Jung Li, Tai-Ling Chao, Sui-Yuan Chang, Shih-Chung Chang

**Affiliations:** 1 Department of Biochemical Science and Technology, College of Life Science, National Taiwan University, Taipei, 106 Taiwan; 2 Department of Clinical Laboratory Sciences and Medical Biotechnology, College of Medicine, National Taiwan University, Taipei, 100 Taiwan; 3 Department of Laboratory Medicine, College of Medicine, National Taiwan University Hospital, National Taiwan University, Taipei, 100 Taiwan; 4 Center of Biotechnology, National Taiwan University, Taipei, 106 Taiwan

**Keywords:** SARS-CoV-2, Spike protein, Epitope peptide, Broadly neutralizing antibody (bnAb), Humoral immunity, Cellular immunity

## Abstract

**Abstract:**

Our previous study has found that monoclonal antibodies targeting a conserved epitope peptide spanning from residues 1144 to 1156 of SARS-CoV-2 spike (S) protein, namely S(1144–1156), can broadly neutralize all of the prevalent SARS-CoV-2 strains, including the wild type, Alpha, Epsilon, Delta, and Gamma variants. In the study, S(1144–1156) was conjugated with bovine serum albumin (BSA) and formulated with Montanide ISA 51 adjuvant for inoculation in BALB/c mice to study its potential as a vaccine candidate. Results showed that the titers of S protein-specific IgGs and the neutralizing antibodies in mouse sera against various SARS-CoV-2 variants, including the Omicron sublineages, were largely induced along with three doses of immunization. The significant release of IFN-γ and IL-2 was also observed by ELISpot assays through stimulating vaccinated mouse splenocytes with the S(1144–1156) peptide. Furthermore, the vaccination of the S(1143–1157)- and S(1142–1158)-EGFP fusion proteins can elicit more SARS-CoV-2 neutralizing antibodies in mouse sera than the S(1144–1156)-EGFP fusion protein. Interestingly, the antisera collected from mice inoculated with the S(1144–1156) peptide vaccine exhibited better efficacy for neutralizing Omicron BA.2.86 and JN.1 subvariants than Omicron BA.1, BA.2, and XBB subvariants. Since the amino acid sequences of the S(1144–1156) are highly conserved among various SARS-CoV-2 variants, the immunogen containing the S(1144–1156) core epitope can be designed as a broadly effective COVID-19 vaccine.

**Key points:**

*• Inoculation of mice with the S(1144–1156) peptide vaccine can induce bnAbs against various SARS-CoV-2 variants.*

*• The S(1144–1156) peptide stimulated significant release of IFN-γ and IL-2 in vaccinated mouse splenocytes.*

*• The S(1143–1157) and S(1142–1158) peptide vaccines can elicit more SARS-CoV-2 nAbs in mice.*

## Introduction

Severe acute respiratory syndrome coronavirus 2 (SARS-CoV-2) has caused the global pandemic of coronavirus disease 2019 (COVID-19), resulting in more than seven million confirmed deaths according to the data reported by the World Health Organization (WHO) (https://data.who.int/dashboards/covid19/). In the past 4 years, the major prevalent SARS-CoV-2 strains have been iterated from the original wild type to the most recent Omicron variants (Hattab et al. 2024; Sah et al. 2023). It is also noted that the first generation of the COVID-19 vaccines, which was mainly designed on the basis of the wild-type strain, has been markedly losing the effectiveness against the Omicron variant (Guo et al. 2023; Pather et al. 2023). The administration of the first-generation COVID-19 vaccine has effectively slowed the spread of SARS-CoV-2, but has not fully eradicated it. For healthcare workers, the elderly, children, and people with weakened immune systems, it is highly recommended to receive regular boosters with newly developed monovalent or bivalent COVID-19 vaccines in order to obtaining adequate immunity against emerging SARS-CoV-2 variants or reducing the risk of illness and death (Arbel et al. 2023; Chi et al. 2022; Plumb et al. 2024; Urschel et al. 2024). In terms of the high mutation rates of the RNA viruses, it is time-consuming and cost-ineffective to develop a new version of SARS-CoV-2 vaccine in response to the frequent breakthrough infections caused by the emerging viral variants. Therefore, it is imperative to accelerate the development of a broad-spectrum or universal COVID-19 vaccines (Li et al. 2022b).

It has been demonstrated that vaccines composed of different SARS-CoV-2 strains may induce broader immunity against emerging variants compared to the homologous vaccine administration (Zhao et al. 2022). Assembling key immunogens from different SARS-CoV-2 strains into a chimeric protein can elicit cross-protective antibodies (Liang et al. 2022; Wu et al. 2022; Xu et al. 2022). Furthermore, it has been found that many SARS-CoV-2 neutralizing antibodies target the conserved neutralizing epitopes in the spike (S) protein, particularly in the S1-RBD and S2 regions (Li and Chang 2023; Yuan et al. 2020). However, while RBD subunit vaccines are immunogenic, their efficacy against SARS-CoV-2 variants is suboptimal due to its high mutation rates at the key residues that are recognized by neutralizing antibodies (Dai et al. 2020; He et al. 2023; Yang et al. 2020). Notably, S2 has been identified as a conserved region among SARS-CoV-2 variants for engaging in the process of membrane fusion between virus and host cell (Shah et al. 2021; Walls et al. 2020). Leveraging these conserved neutralizing epitopes found in S2 represents promising opportunities for the development of universal vaccines (Li et al. 2021). Our group has demonstrated that mouse monoclonal antibodies (mAbs) S2-4D, S2-5D, S2-8D, and S2-4A can broadly neutralize various SARS-CoV-2 strains, including wild type, Alpha, Epsilon, Delta, and Gamma variants, through recognizing an epitope peptide spanning from residues 1144 to 1156 on the SARS-CoV-2 S protein, namely S(1144–1156), and blocking the S protein-triggered membrane fusion (Li et al. 2022a). Notably, the antibody repertoire against the S(1144–1156) epitope peptide can also be elicited through natural SARS-CoV-2 infection in COVID-19 convalescent patients (Li et al. 2022a). Therefore, it is considered that the S(1144–1156) might be an important epitope peptide for eliciting humoral immune responses and SARS-CoV-2 neutralizing antibodies. In the study, the epitope peptide S(1144–1156) was conjugated with bovine serum albumin (BSA), namely S(1144–1156)_BSA, for immunization in BALB/c mice to examine the induction levels of the S protein-specific IgGs and the virus neutralizing activities against various SARS-CoV-2 variants. The antibody profile induced by immunization of S(1144–1156)_BSA was determined by alanine scanning mutagenesis and western blot (WB) analysis. The resulting cellular immune responses following immunization were measured by ELISpot assays via stimulating mouse splenocytes with the S(1144–1156) peptide for measuring the secretion of IFN-γ and IL-2. In addition, a series of S(1144–1156)-extended enhanced green fluorescent protein (EGFP)-fusion proteins were also used as the immunogens in the animal experiments to investigate whether the S(1144–1156)-extended epitope peptides containing one or two N- and C-terminal extra residues can also elicit neutralizing antibodies against various SARS-CoV-2 variants. Subsequently, the antisera were used to examine the virus neutralizing efficacy against various Omicron subvariants for better understanding the potential of the S(1144–1156) epitope peptide as a broad-spectrum COVID-19 vaccine.

## Materials and methods

### Peptide synthesis

The peptide sequence ELDSFKEELDKYF of S(1144–1156) was synthesized with an additional C-terminal cysteine (Yao-Hong Biotechnology Inc., New Taipei City, Taiwan) and then conjugated to bovine serum albumin (BSA) for producing the S(1144–1156)_BSA immunogen in the correct orientation.

### Preparation of S(1144–1156)-extended EGFP-fusion proteins

The cDNA sequences encoding the S(1144–1156), S(1143–1157), or S(1142–1158) peptide were inserted at the 5′-end of the EGFP cDNA and cloned into the pET28a plasmid (Merck Millipore, Burlington, MA, USA) to express a series of S(1144–1156)-extended EGFP fusion proteins, referred to as 13-EGFP, 15-EGFP, and 17-EGFP. The produced pET28a plasmids were transformed into *Escherichia coli* BL21(DE3) competent cells (Merck Millipore, Burlington, MA, USA). Protein expression was induced by adding 1 mM isopropyl β-D-1-thiogalactopyranoside (IPTG) to the culture medium and incubating at 37 °C for 4 h. Bacterial cells were homogenized in phosphate-buffered saline (PBS), and the supernatants from the cell lysates were loaded onto a HisTrap FF column (Cytiva, Marlborough, MA, USA) for purification using a linear gradient of 20–250 mM imidazole. The purified S(1144–1156)-extended EGFP fusion proteins were buffer-exchanged with PBS using a PD-10 column (Cytiva, Marlborough, MA, USA).

### Preparation of the recombinant SARS-CoV-2 S protein

The pSecTag2A vector (Thermo Fisher Scientific, Waltham, MA USA), which contains the cDNA encoding the recombinant SARS-CoV-2 S protein (GenBank accession no. BCN86353.1) with a C-terminal hexa-histidine tag (His-tag), was transfected into Expi293F cells using the Expi293 Expression System following the manufacturer’s instructions (Thermo Fisher Scientific, Waltham, MA USA). After incubating cells at 37 °C in the 8% CO_2_ incubator for 5 days, the culture medium was collected for the purification of the recombinant S protein using the HisTrap FF column (Cytiva, Marlborough, MA, USA) as described previously (Lai et al. 2022).

### Inoculation of mice with the S(1144–1156)-based antigens

Seven-week-old female BALB/c mice (*n* = 5) bought from the Association for Assessment and Accreditation of Laboratory Animal Care International (AAALAC)-accredited National Laboratory Animal Center Taiwan were inoculated with 50 µg of BSA or S(1144–1156)_BSA formulated with 50 µL of Montanide ISA™ 51 adjuvant (ISA 51, Seppic, La Garenne-Colombes, France) through subcutaneous injection on days 0, 14, and 28. For inoculation of EGFP or the S(1144–1156)-extended EGFP-fusion proteins (13-EGFP, 15-EGFP, and 17-EGFP), 7-week-old female BALB/c mice (*n* = 3) were immunized with 50 µg of EGFP or the indicated EGFP-fusion proteins mixed with 50 µL of Freund’s adjuvant through intraperitoneal injection on days 0, 14, 28, and 42. Mouse antiserum was collected from the submandibular vein 2 weeks after each inoculation. The antiserum samples were heated at 56 °C for 30 min, filtered through a 0.22-μm membrane disc, and stored at 4 °C until use.

### ELISA

Mouse antiserum, diluted 1:1,000 in PBS, was added to a 96-well plate pre-coated with 100 ng of SARS-CoV-2 S protein and incubated at 37 °C for 1 h. After three washing steps with PBST (PBS containing 0.05% Tween 20), the horseradish peroxidase (HRP)-conjugated secondary antibody (5450–0011, SeraCare, Milford, MA, USA), diluted 1:50,000 in PBS, was added to probe the primary antibody. The substrate solution containing hydrogen peroxide and 3,3′,5,5′-tetramethylbenzidine (BD Bioscience, Franklin Lakes, NJ, USA) was then used for signal detection. The color development was stopped with a solution of 2 N H_2_SO_4_. Absorbance was measured at 450 nm using the Multiskan FC Microplate Photometer (Thermo Fisher Scientific, Waltham, MA, USA) to record the results.

### Plaque reduction neutralization test (PRNT)

All PRNT assays were conducted with the procedures described previously (Li et al. 2022a). The SARS-CoV-2 variants applied in the study include the wild-type strain hCoV-19/Taiwan/NTU13/2020 (GISAID: EPI_ISL_422415), Alpha hCoV-19/Taiwan/NTU49/2021 (GISAID: EPI_ISL_1010728), Delta hCoV-19/Taiwan/NTU92/2021 (GISAID: EPI_ISL_3979387), Omicron BA.1 hCoV-19/Taiwan/NTU128/2021 (GISAID: EPI_ISL_11050301), Omicron BA.2 hCoV-19/Taiwan/NTU142/2022 (GISAID: EPI_ISL_13105945), Omicron XBB hCoV-19/Taiwan/NTU293/2023 (GISAID: EPI_ISL_18278880), Omicron BA.2.86 hCoV-19/Taiwan/NTU295/2024 (GISAID: EPI_ISL_19061462), and Omicron JN.1 hCoV-19/Taiwan/NTU299/2024 (GISAID: EPI_ISL_19061466). Briefly, the mouse antiserum was incubated with the viral solution at 37 °C for 1 h before being added to a monolayer of Vero E6 cells cultured at 37 °C in the 5% CO_2_ incubator. After 1 h of incubation and the subsequent washing steps with PBS, the cells were overlaid with 2% methylcellulose in the fetal bovine serum (FBS)-supplemented Dulbecco’s Modified Eagle Medium (DMEM) (Thermo Fisher Scientific, Waltham, MA USA). Five days later, the cells were fixed with 10% formaldehyde and stained with 0.5% crystal violet to observe the plaque formation. The dilution ratio of the antiserum that resulted in a 50% reduction in the number of plaques compared to the virus-only control was designated as PRNT50.

### Alanine scanning mutagenesis

The pET30a plasmid was bought from Merck Millipore (Burlington, MA, USA). The pET30a-sfGFP-S(1042–1167)-His plasmid served as the template for alanine scanning site-directed mutagenesis. Specifically, residues 1142–1158 of the sfGFP-S(1042–1167)-His were individually substituted with alanine using the conventional PCR method as previously described (Li et al. 2022a). *E. coli* BL21(DE3) competent cells were used to produce a series of sfGFP-S(1042–1167)-His mutants. Protein expression was induced by adding 1 mM of IPTG to the culture medium and incubating at 37 °C for 4 h. The bacterial cells were then homogenized in PBS using a cell disruptor to obtain the whole cell lysates.

### WB analysis

Samples were separated using the sodium dodecyl sulfate–polyacrylamide gel electrophoresis (SDS-PAGE) under the reduction condition and then transferred to a polyvinylidene difluoride (PVDF) membrane (IPVH85R, Merck Millipore, Burlington, MA, USA). The PVDF membrane was blocked with 5% non-fat milk in PBST for 30 min and subsequently incubated with either the mAb sample or mouse antiserum (1:5,000 in blocking buffer) at room temperature for 1 h. After washing with PBST, the HRP-conjugated secondary antibody (5450–0011, SeraCare, Milford, MA, USA) was added to probe the primary antibody. The chemiluminescent substrate (VisGlow, Visual Protein Biotechnology, Taipei, Taiwan) was used to develop the WB signal, which was captured by the UVP BioSpectrum Imaging System (Upland, CA, USA).

### ELISpot analysis

The collected splenocytes were filtered with a 100-µm filter membrane and centrifuged at 300 × *g* for 10 min for removal of the supernatant. Splenocytes were incubated with 2 mL of red blood cell lysis buffer for 2 min and resuspended with 10 mL of DMEM containing 10% FBS. Mouse IFN-γ and IL-2 ELISpot Kits (R&D Systems, Minneapolis, MN, USA) were used to perform the ELISpot analysis according to the manufacturer’s instructions. In brief, 200 µL of culture medium and 100 µL of 5 × 10^5^ splenocytes were added to each well of the 96-well plate and incubated at room temperature for 20 min. For stimulating the antigen-specific immune response, 100 µL of the synthetic S(1144–1156) peptide (10 µg/mL) was added to the splenocyte culture. After 40 h of incubation at 37 °C in the 5% CO_2_ incubator, the culture medium was removed and the wells were washed with PBST. Anti-IFN-γ or anti-IL-2 detection antibody (100 µL) was added to the wells and incubated at 4 °C overnight. After three washing steps using PBST, 100 µL of streptavidin–alkaline phosphatase solution was added to the wells and incubated at room temperature for 2 h. For signal detection, 100 µL of BCIP/NBT substrate was used for color development. After 1 h, the solution was removed and the wells were rinsed with deionized water. The 96-well plate was then air-dried at room temperature for 90 min. The blue-black colored precipitates form at the sites of cytokine localization and appear as spots. The ELISpot result was examined by the ImmunoSpot S6 Universal Analyzer (Cellular Technology Limited, Cleveland, OH, USA) to calculate the numbers of spots.

### Statistical analysis

Statistical analysis was performed by using the GraphPad Prism 10 software (GraphPad Software, Boston, MA, USA). Data are presented as means ± standard deviations (SD). The statistical significance was calculated with unpaired Student’s *t*-test. Triple asterisk (***) represents *P* ≤ 0.001.

## Results

### Immunization of S(1144–1156)_BSA in mice for eliciting broadly neutralizing antibodies against SARS-CoV-2 variants

**Fig. 1:**
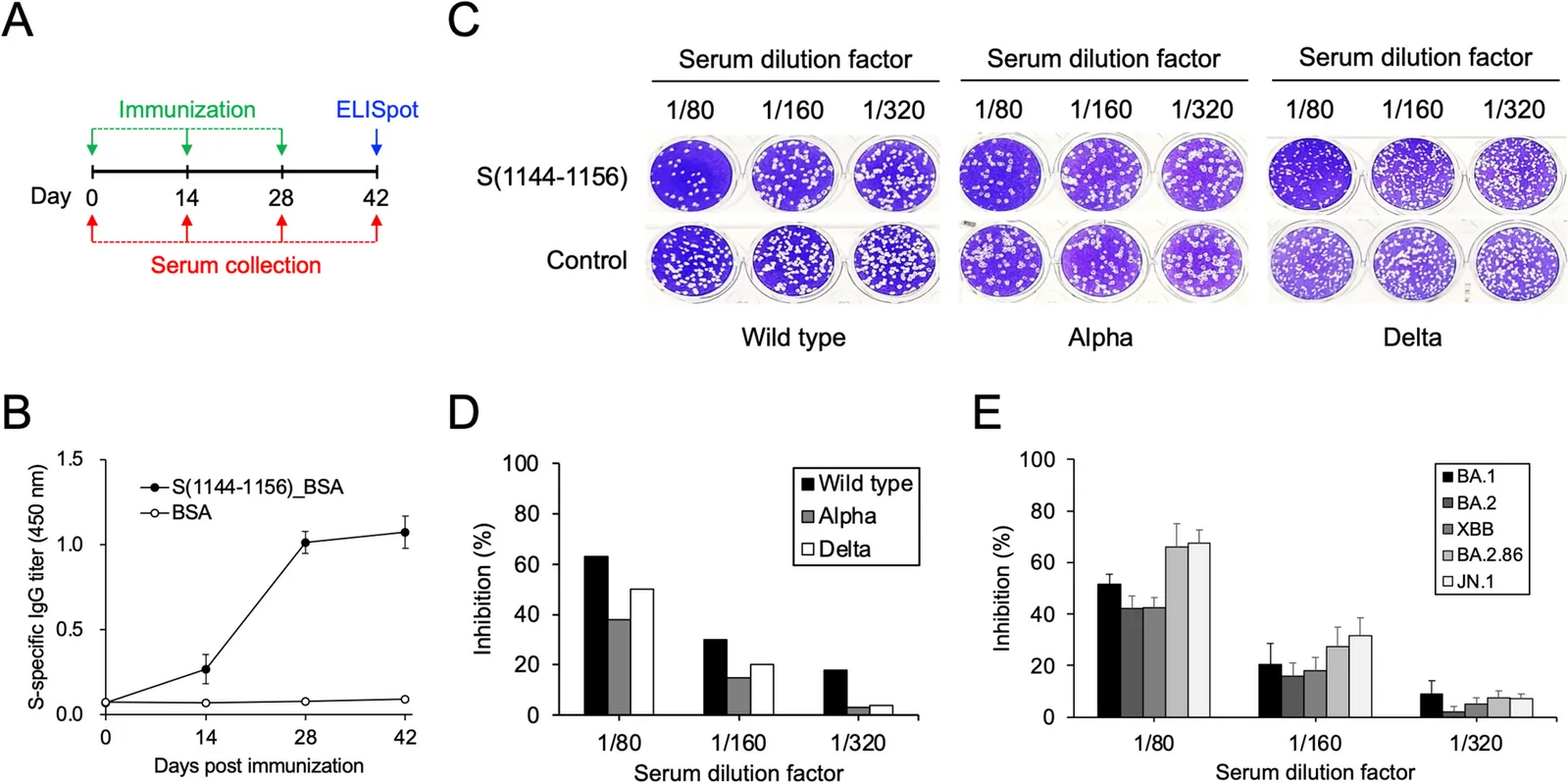
S(1144–1156) conjugated with BSA can induce broadly neutralizing antibodies against SARS-CoV-2 wild type, Alpha, Delta, and Omicron variants. **A** Three doses of S(1144–1156)_BSA (50 μg per dose) were inoculated in BALB/c mice in a 2-week interval. Antisera were collected before beginning the animal experiment and 2 weeks post each immunization. Splenocytes were collected 2 weeks post receiving the third dose of S(1144–1156)_BSA for ELISpot analysis. **B** The specific IgG titers of the collected antisera against SARS-CoV-2 S protein (black solid circles) and BSA (white open circles) were measured by ELISA. Data represent means ± SD (*n* = 5). **C** The collected antisera were serially diluted as indicated in the figure and then incubated with various SARS-CoV-2 variants, including wild-type strain, Alpha variant, or Delta variant, for performing the PRNT assays. **D** Plaques formed in the previous PRNT assays were counted and the inhibition of the plaque formation was calculated by comparing to the results by using the antisera collected from the control animal group inoculated with BSA (control). **E** The collected antisera were serially diluted as indicated in the figure and then incubated with various Omicron subvariants, including BA.1, BA.2, XBB, BA.2.86, and JN.1, for performing the PRNT assays. Data represent means ± SD (*n* = 5)

S(1144–1156) is an antigenic peptide recognized by a specific group of SARS-CoV-2 broadly neutralizing mAbs and human serum antibodies of COVID-19 convalescent patients (Li et al. 2022a). Therefore, S(1144–1156) was conjugated with BSA and adjuvanted with ISA 51 for immunization in BALB/c mice to examine its effectiveness as a vaccine candidate (Fig. 1A). The results showed that as the doses of S(1144–1156)_BSA administered to mice increased, the induction of the S protein-specific IgGs was markedly observed (Fig. 1B). The mouse antisera collected on day 42 since initial immunization (2 weeks post third immunization) were also subjected to the PRNT assay (Fig. 1C). It is found that while the antisera collected from the S(1144–1156)_BSA group were diluted 80-fold, they can exhibit 60%, 40%, and 50% neutralizing activities against SARS-CoV-2 wild type, Alpha, and Delta variants, respectively (Fig. 1D). More importantly, we found that the elicited antisera can neutralize all of the Omicron BA.1, BA.2, XBB, BA.2.86, and JN.1 subvariants (Fig. 1E). Interestingly, the antisera neutralized BA.2.86 and JN.1 with higher efficacy than neutralized BA.1, BA.2, and XBB (Fig. 1E).

### The antibody profile induced by immunization of S(1144–1156)_BSA in mice

**Fig. 2:**
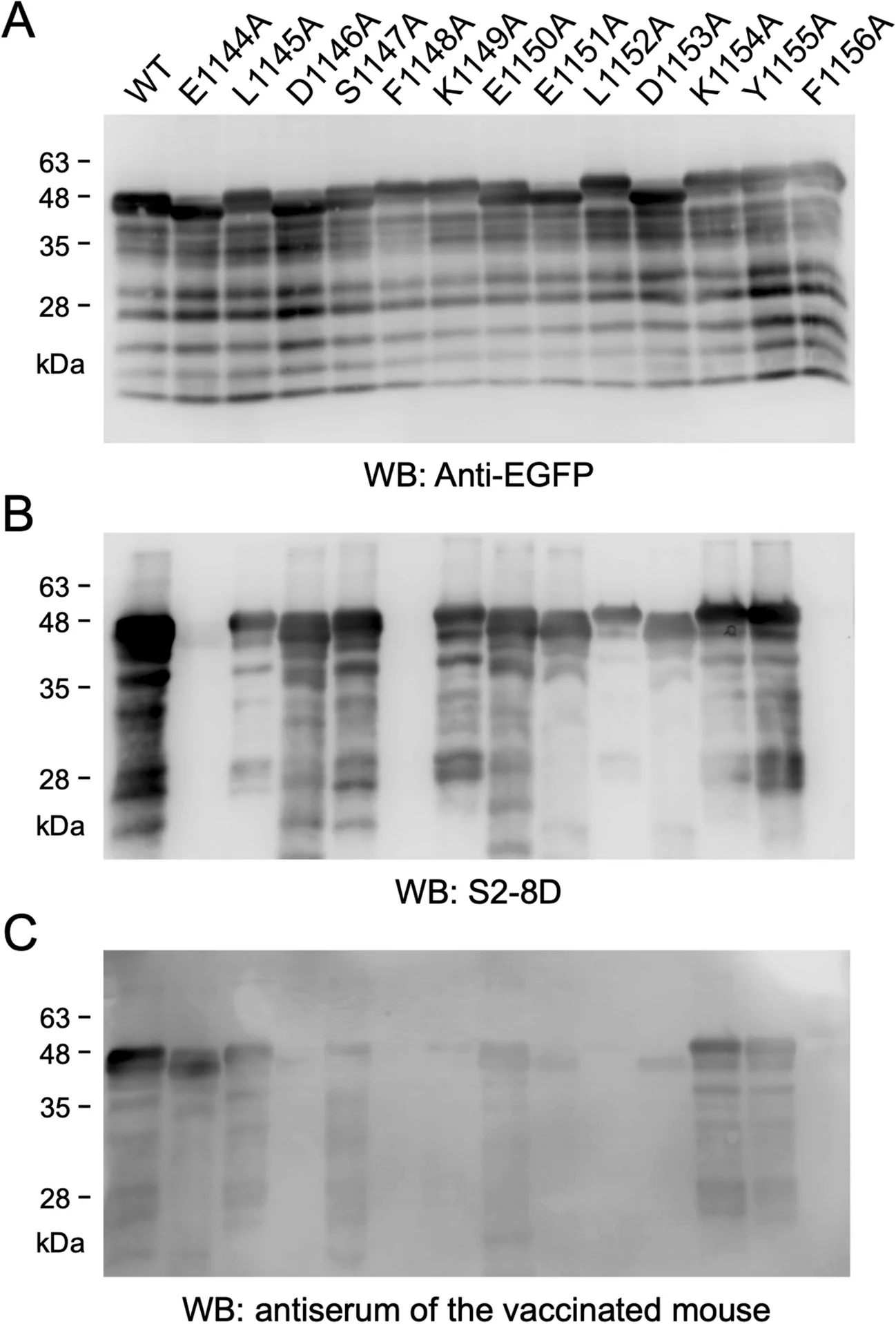
Epitope mapping of the antiserum collected from the BALB/c mouse inoculated with S(1144–1156)_BSA. The *E. coli*-expressed sfGFP-S(1042–1167) fusion proteins containing a series of individual alanine substitutions at residues 1144–1156 were subjected to SDS-PAGE and WB analysis with **A** anti-EGFP mAb, **B** S2-8D, and **C** antiserum collected from the BALB/c mouse which has received three doses of S(1144–1156)_BSA

The alanine scanning mutagenesis was applied to generate a series of sfGFP-S(1042–1167) mutants which were then separated by SDS-PAGE for WB analysis with anti-GFP mAb, S2-8D (an SARS-CoV-2 neutralizing mAb), and antiserum derived from the S(1144–1156)_BSA-immunized mouse (Fig. 2). As we have shown previously (Li et al. 2022a), alanine mutations at residues 1144, 1148, and 1156 of sfGFP-S(1042–1167) may greatly reduce S2-8D binding to the target antigen (Fig. 2B), indicating that these three residues are the key antigenic determinants of S2-8D. Alanine mutations at the nearby residues 1145 and 1152 may also slightly interrupt S2-8D binding to sfGFP-S(1042–1167) (Fig. 2B). Notably, the antiserum derived from the S(1144–1156)_BSA-immunized mouse exhibited a broader antibody profile than that of S2-8D and the binding sites were distributed throughout the epitope peptide spanning from residues 1144 to 1156 (Fig. 2C). Like S2-8D, the antiserum showed lower binding ability against sfGFP-S(1042–1167) containing the E1144A, F1148A, L1152A, or F1156A mutation. Moreover, if the sfGFP-S(1042–1167) antigen contained the D1146A, S1147A, K1149A, E1150A, E1151A, D1153A, or Y1155A mutation, it was not properly recognized by the elicited antiserum (Fig. 2C). Interestingly, the sfGFP-S(1042–1167) containing the E1144A mutation was not largely detected by the elicited antiserum, implying that immunization of S(1144–1156) epitope peptide did not efficiently induce antibodies which interacted with residue E1144 (Fig. 2C, lane 2).

### The adaptive cellular immunity acquired by vaccination of ISA 51-adjuvanted S(1144–1156)_BSA

**Fig. 3:**
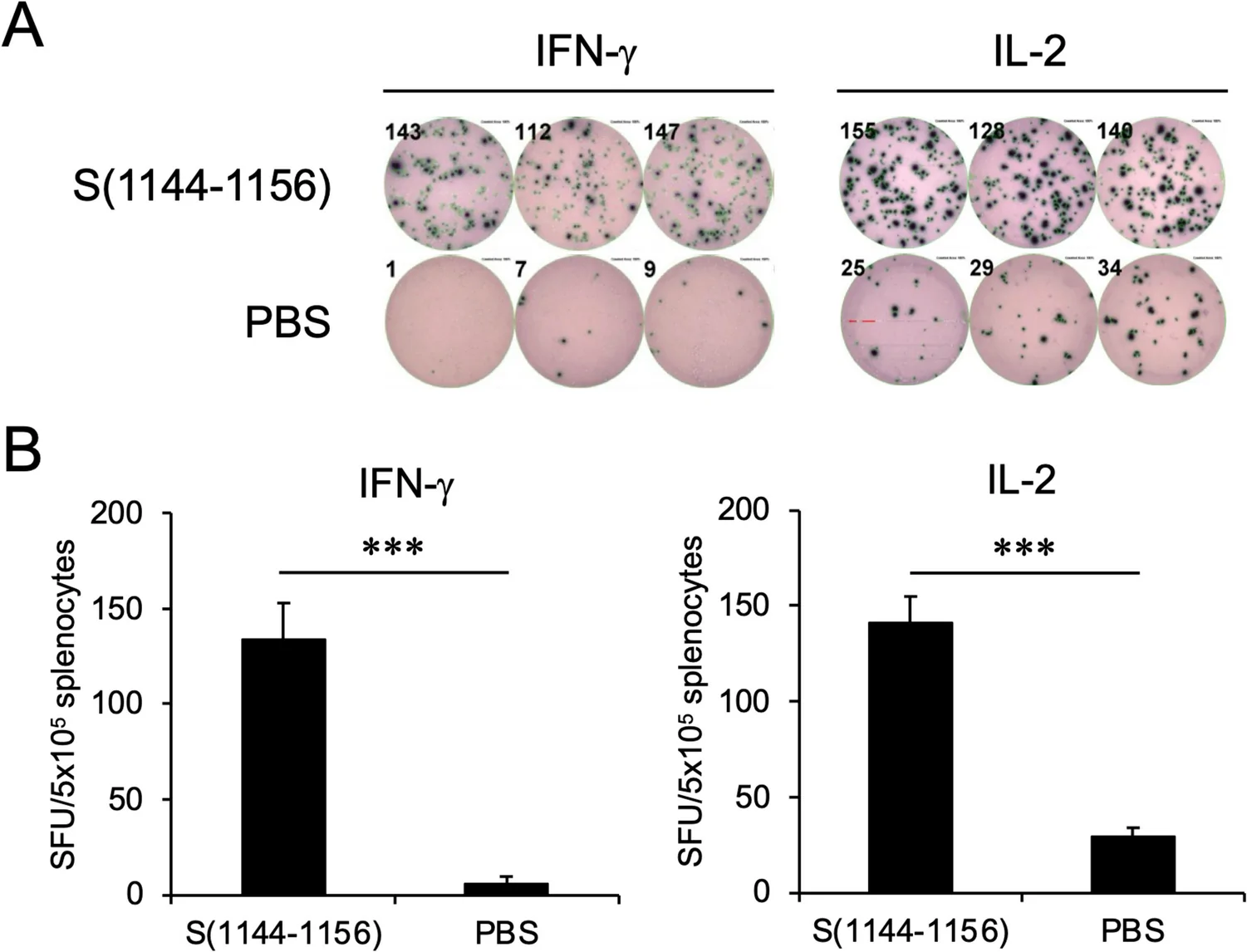
S(1144–1156)_BSA formulated with ISA 51 adjuvant can induce cellular immunity and significant release of IFN-γ and IL-2 in splenocytes. **A** The splenocytes collected from the BALB/c mice which have received three doses of S(1144–1156)_BSA were applied in the ELISpot analysis. The synthetic S(1144–1156) peptide (upper panels) or PBS (lower panels) was added to the culture medium for stimulating the secretion of IFN-γ and IL-2. Each panel contains three independent experiments. The counting numbers of the forming spots were shown in the upper left of the wells. **B** The numbers of spots were calculated by the ImmunoSpot S6 Universal Analyzer and the ELISpot analysis readout was expressed as spot forming units (SFU) per 10^5^ cells. Data represent means ± SD (*n* = 3). The statistical significance was calculated with unpaired *t*-test, ***P < 0.001, S(1144–1156) vs PBS

To investigate whether the adaptive cellular immunity was acquired by vaccination of the ISA 51-adjuvanted S(1144–1156)_BSA, the splenocytes obtained from the BALB/c mice which have received three doses of the ISA 51-adjuvanted S(1144–1156)_BSA were used to perform the ELISpot analysis (Fig. 1A). After stimulating the splenocytes with S(1144–1156) peptide, the release of IFN-γ and IL-2 were captured by the anti-IFN-γ and anti-IL-2 antibodies on the bottom of the PVDF-backed microplate, and then detected by the alkaline phosphatase-based color developing system. The results showed that a lot of blue-black precipitates formed at the sites of cytokine localization and appeared as spots (Fig. 3A), which were further counted with an automated ELISpot reader system. It is clearly observed that IFN-γ and IL-2 were specifically stimulated by S(1144–1156) peptide and significantly released by the splenocytes obtained from the ISA 51-adjuvanted S(1144–1156)_BSA-immunized BALB/c mice (Fig. 3B).

### Eliciting SARS-CoV-2 neutralizing antibody by immunization of mice with the S(1144–1156)-extended EGFP

**Fig. 4:**
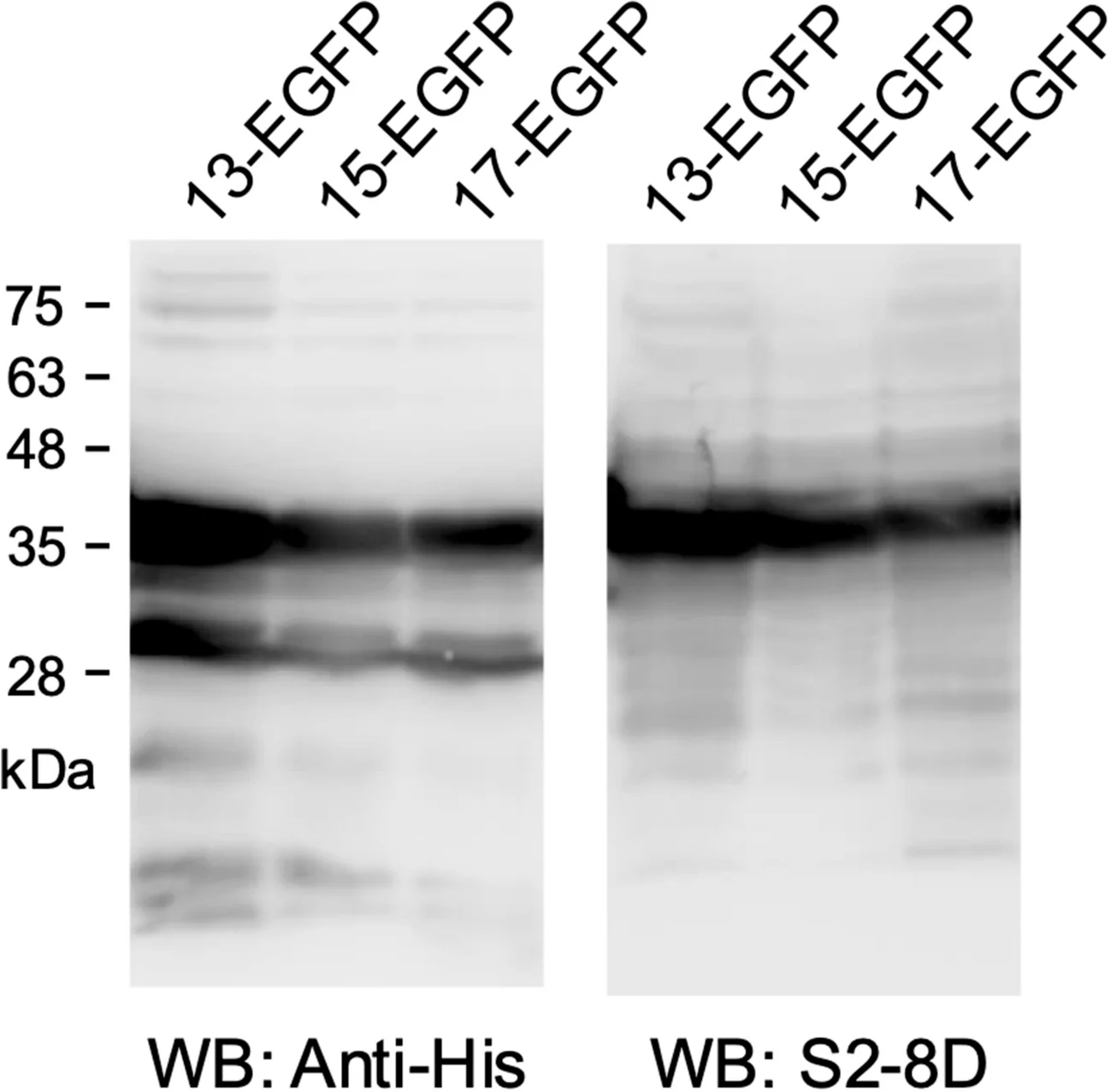
The S2-8D can recognize the S(1144–1156)-extended EGFP-fusion proteins. The *E. coli*-expressed S(1144–1156)-extended EGFP-fusion proteins (13-EGFP, 15-EGFP, and 17-EGFP) were analyzed by SDS-PAGE and then subjected to WB analysis with anti-His mAb or S2-8D, respectively

**Fig. 5:**
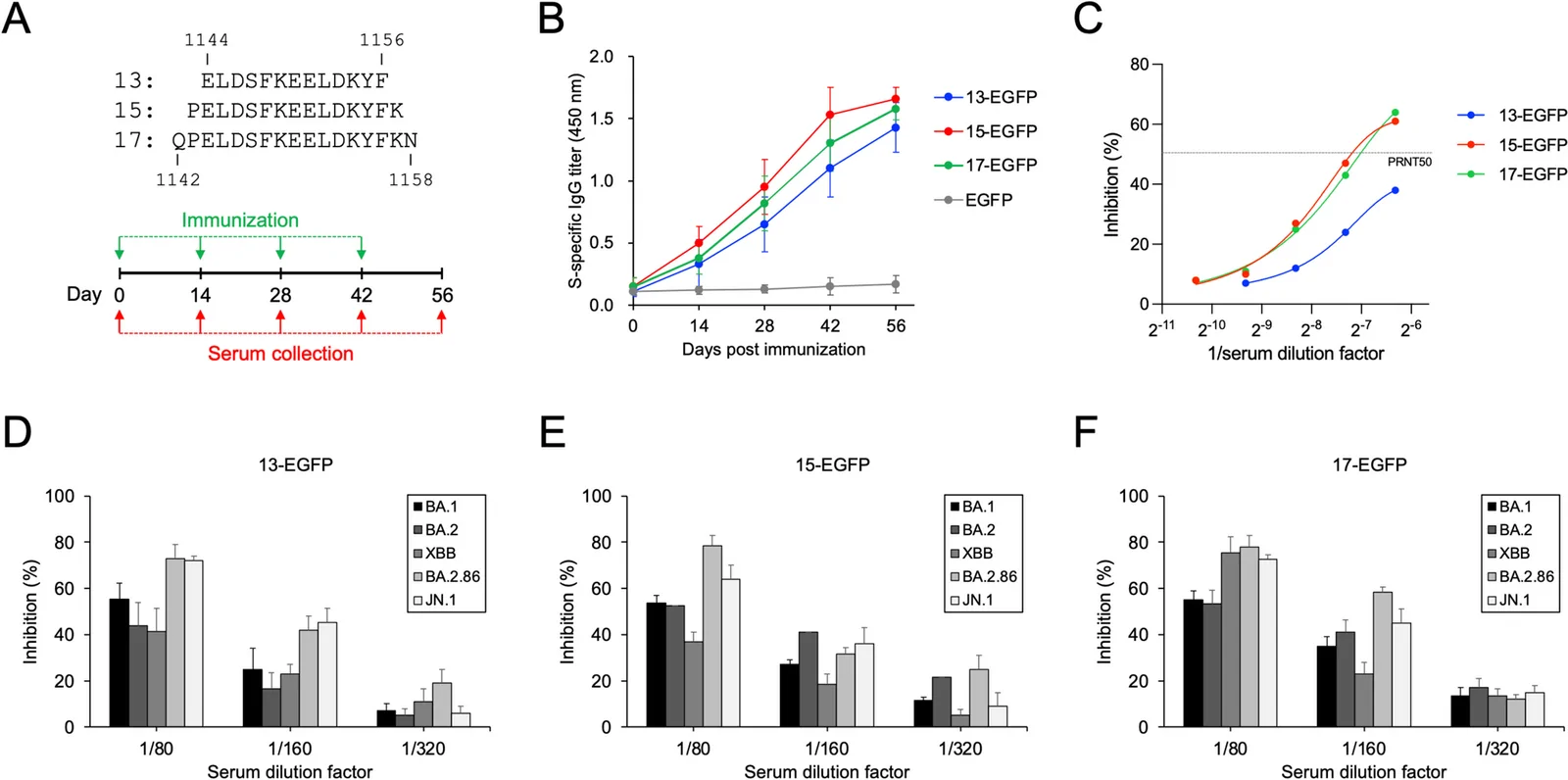
The S(1144–1156)-extended EGFP-fusion proteins can induce SARS-CoV-2 neutralizing antibodies. **A** Four doses of the S(1144–1156)-extended EGFP-fusion proteins (13-EGFP, 15-EGFP, and 17-EGFP) containing the indicated amino acid sequences shown in the upper panel were inoculated in BALB/c mice in a 2-week intervals. Antiserum was collected five times as indicated in the schematic diagram. **B** The specific IgG titers of the collected antisera against SARS-CoV-2 S protein were measured by ELISA. Data represent means ± SD (*n* = 3). **C** The collected antisera were serially diluted and then incubated with SARS-CoV-2 wild-type strain for performing the PRNT assays. The inhibition of the plaque formation was calculated by comparing to the results using the antisera collected from the control animal group inoculated with EGFP. The gray dotted line represents the PRNT50. The collected antisera from the 13-EGFP group (**D**), the 15-EGFP group (**E**), or the 17-EGFP group (**F**) were also serially diluted and used for performing the PRNT assays against Omicron BA.1, BA.2, XBB, BA.2.86, and JN.1 subvariants

To study whether the longer peptides which also contain the core sequence of S(1144–1156) can elicit higher levels of SARS-CoV-2 neutralizing antibody, the S(1144–1156)-extended epitope peptides containing one or two N- and C-terminal extra residues were expressed as the recombinant EGFP-fusion proteins (namely 13-EGFP: S(1144–1156)-EGFP, 15-EGFP: S(1143–1157)-EGFP, and 17-EGFP: S(1142–1158)-EGFP). The 13-EGFP, 15-EGFP, and 17-EGFP proteins can be functionally recognized by S2-8D (Fig. 4), indicating that the core epitope peptide S(1144–1156) of these fusion protein antigens normally exposed to the surface of EGFP. Four doses of the Freund’s adjuvant-supplemented 13-EGFP, 15-EGFP, and 17-EGFP were then inoculated into BALB/c mice in a 2-week interval (Fig. 5A). The antisera collected at the beginning of the animal experiment and 2 weeks post each immunization were subjected to ELISA for determination of the S protein-specific IgG titer. The results showed that the S protein-specific IgG titers of the 15-EGFP and 17-EGFP groups were slightly higher than the titers of the 13-EGFP group (Fig. 5B). The PRNT assays also showed that the SARS-CoV-2 neutralizing activities of the antisera collected from the 15-EGFP and 17-EGFP groups were higher than that of the 13-EGFP group (Fig. 5C), suggesting that the S(1144–1156)-extended epitope peptides can elicit more neutralizing antibodies. In addition, the antisera collected from the 13-EGFP, 15-EGFP, and 17-EGFP groups exhibited neutralizing activities against all of the Omicron subvariants. Interestingly, they performed better efficacy for neutralizing BA.2.86 and JN.1 than BA.1, BA.2, and XBB (Fig. 5D and E).

### The antibody profile induced by immunization of the S(1144–1156)-extended EGFP in mice

**Fig. 6:**
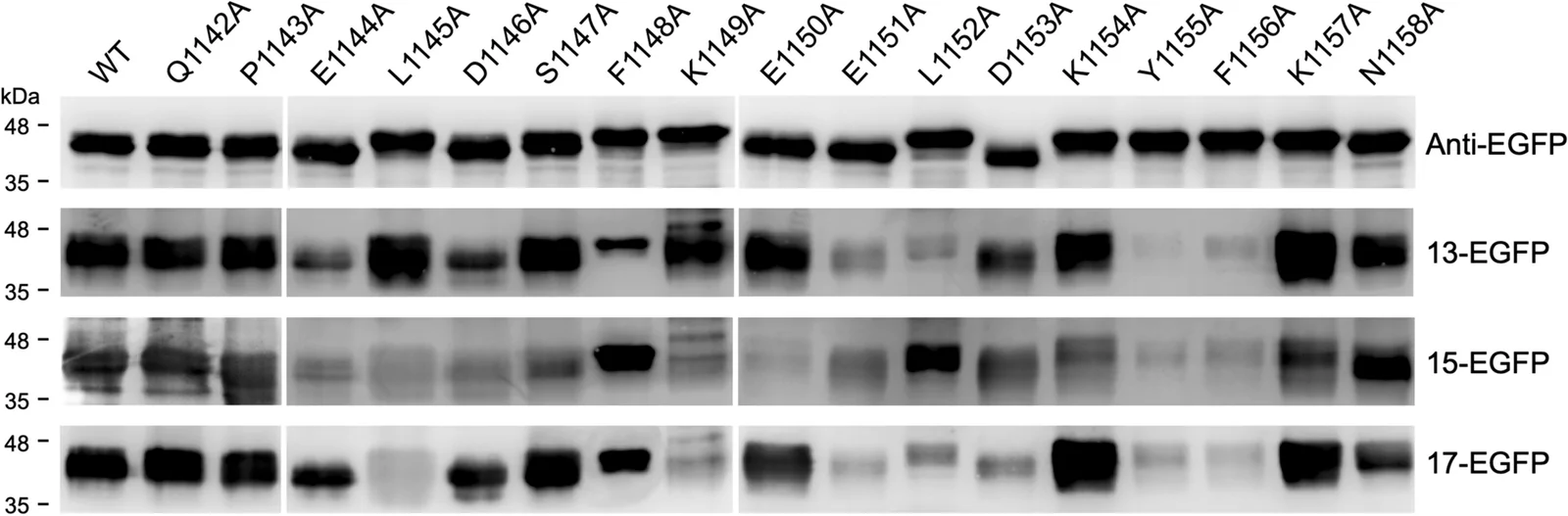
Epitope mapping of the antisera collected from the BALB/c mice inoculated with the S(1144–1156)-extended EGFP-fusion proteins. The *E. coli*-expressed sfGFP-S(1042–1167) fusion proteins containing a series of individual alanine substitutions at residues 1142–1158 were subjected to SDS-PAGE and WB analysis with anti-EGFP mAb and the antisera collected from the BALB/c mice which have received four doses of the Freund’s adjuvant-supplemented S(1144–1156)-extended EGFP-fusion proteins (13-EGFP, 15-EGFP, and 17-EGFP), respectively

The antisera collected from BALB/c mice which have been inoculated with the S(1144–1156)-extended EGFP were also subjected to the epitope mapping experiments by using the alanine scanning mutagenesis as described previously. The results showed that the sfGFP-S(1042–1167) containing an alanine mutation at residue 1144, 1146, 1148, 1151, 1152, 1153, 1155, or 1156 cannot be properly recognized by the antiserum of the 13-EGFP group (Fig. 6). The sfGFP-S(1042–1167) containing an alanine mutation at residues 1144, 1145, 1146, 1147, 1149, 1150, 1151, 1153, 1154, 1155, or 1156 cannot be properly recognized by the antiserum of the 15-EGFP group (Fig. 6). Similar with the results of 13-EGFP group, the sfGFP-S(1042–1167) containing an alanine mutation at residues 1144, 1145, 1146, 1148, 1149, 1151, 1152, 1153, 1155, or 1156 cannot be properly recognized by the antiserum of the 17-EGFP group (Fig. 6). It is clear that the key residues recognized by these antiserum samples are all localized within residues 1144–1156. Among them, residues 1144, 1146, 1151, 1152, 1153, 1155, and 1156 are the major antigenic determinants recognized by these three antiserum samples. Interestingly, an alanine mutation at residue 1145 or 1149 did not inhibit binding by the antiserum sample collected from the 13-EGFP group, whereas it greatly reduced binding by the antiserum samples collected from the 15-EGFP group and 17-EGFP group (Fig. 6), indicating that the 13-EGFP group contains broader antibody profiles covering more amino acid residues. The results imply that immunization of 13-EGFP in mice can elicit more broad-spectrum antibodies than immunization of 15-EGFP and 17-EGFP.

## Discussion

SARS-CoV-2 has been continually mutating with more genetic variability and becoming more transmissible with a higher mean reproduction number (R0) as they spread worldwide (Liu and Rocklov 2021, 2022; Manathunga et al. 2023). Numerous studies have shown that the effectiveness of approved vaccines and the neutralizing potency of therapeutic antibodies were profoundly reduced to a feeble level while facing the breakthrough infection with the Omicron variant (Carreno et al. 2022; Kuhlmann et al. 2022; Planas et al. 2022; Tuekprakhon et al. 2022). Furthermore, the approved vaccines and the current strain-specific vaccination strategy are not sufficient for providing broad-spectrum protection against the rapidly mutated SARS-CoV-2 (Ai et al. 2022; Tan et al. 2023). Therefore, it is a big challenge for vaccine developers to find out a long-term solution and invent the broad-spectrum vaccines that are resilient against emerging SARS-CoV-2 variants.

Broadly neutralizing antibodies (bnAbs) have been identified from SARS-CoV-2-infected individuals or COVID-19 vaccine recipients (Ai et al. 2022; Hurlburt et al. 2022; Piepenbrink et al. 2022; Pinto et al. 2021; Zhou et al. 2022), implying that broad-spectrum immunity is possible. We and the others have found a group of SARS-CoV-2 neutralizing antibodies that target a highly conserved epitope in the upstream region of the S protein heptad repeat 2 (HR2) domain (Li and Chang 2023; Wang et al. 2020). Although bnAbs typically target conserved epitopes that are present in all viral variants and vaccines, natural infection and vaccination often fail to induce broad immunity (Tan et al. 2023). It has been found that the peak antibody response to SARS-CoV-2 mRNA vaccination exceeds titers seen in convalescent individuals but comprises a high ratio of non-neutralizing antibodies (Amanat et al. 2021). Therefore, it is crucial to ensure that broad-spectrum vaccines do not induce inappropriate antibodies. In this study, the antisera collected from mice inoculated with three doses of ISA 51-adjuvanted S(1144–1156)_BSA can neutralize all of the SARS-CoV-2 variants tested in the NT assays (Fig. 1C–E), supporting that the conserved neutralizing epitope is a promising broad-spectrum vaccine candidate. Furthermore, the epitope profiles of the S(1144–1156)_BSA vaccine-induced antisera are broader than that of the SARS-CoV-2 S2-specific neutralizing mAb S2-8D (Fig. 2), suggesting that the administration of the epitope peptide vaccine not only induces the specific immune response but also elicits a wide range of neutralizing antibodies which can further improve the vaccine potency. Interestingly, we found that the antisera collected from mice inoculated with S(1144–1156)-EGFP, S(1143–1157)-EGFP, and S(1142–1158)-EGFP exhibited better efficacy for neutralizing BA.2.86 and JN.1 than other Omicron subvariants (Fig. 5D and E). Since Omicron BA.2.86 and JN.1 subvariants contain a P1143L mutation near the S(1144–1156) region, it may change the protein local structure and make the S(1144–1156) epitope more accessible for binding by the S(1144–1156)-specific neutralizing antibodies.

Promoting the T cell immune response and increasing the longevity of memory T and B cell immunity have become the important considerations of vaccine design for combating the frequent SARS-CoV-2 mutations (Chen et al. 2022; Dhawan et al. 2023; Gao et al. 2021; Jain et al. 2021; Moss 2022; Pettini et al. 2022). We observed that inoculation of mice with ISA 51-adjuvanted S(1144–1156)_BSA can elicit very strong T cell response upon stimulating by S(1144–1156) epitope peptide, resulting in significant release of IFN-γ and IL-2 in splenocytes (Fig. 3). These data demonstrated that S(1144–1156) is an immunogenic T cell epitope, but we did not know how the strength of this response compares to the antiviral T cell responses induced by other T cell epitopes. Since the S2 subunit exhibits comparatively lower rates of genetic variability than RBD among different SARS-CoV-2 variants (Olukitibi et al. 2023), likely because the S2 subunit is not directly involved in interaction with the host receptor (Lan et al. 2020), we conclude that S(1144–1156) is a promising peptide vaccine candidate which can induce broadly neutralizing antibodies and promote cellular immunity against SARS-CoV-2 variants.

Vaccination remains to be the most cost-effective strategy for controlling infectious disease outbreaks (Gomez et al. 2021; Senevirathne et al. 2024). The current approach to address the limitations of approved COVID-19 vaccines is to periodically boost with homologous and heterologous vaccines to increase cross-reactive antibody titers and extend the duration of protection (Zhu et al. 2024) Another approach is to incorporate additional key epitopes from emerging SARS-CoV-2 variants into next-generation vaccines for enhancing the possibility of eliciting more cross-protective neutralizing antibodies (Gao et al. 2021). The identification of the conserved bnAb epitopes will support the development of universal vaccines, as they are less likely to lose effectiveness against newly emerging SARS-CoV-2 variants.

## Data Availability

All data associated with this study are included in the paper.
